# The encaged lung: rapidly progressive idiopathic pleurisy

**DOI:** 10.1093/omcr/omy041

**Published:** 2018-08-09

**Authors:** Ivana Castaniere, Roberto Tonelli, Riccardo Fantini, Alessandro Marchioni, Martina Garofalo, Enrico M Clini, Stefania Cerri

**Affiliations:** 1 Department of Medical and Surgical Sciences, University of Modena Reggio Emilia, Modena, Italy; 2 Respiratory Diseases Unit and Centre for Rare Lung Diseases, University Hospital of Modena, Modena, Italy

## Abstract

A 56-year-old, non-smoker male with no exposure, presented with right chest pain and a huge loss in forced vital capacity due to right lung volume reduction with consensual pleural thickening on high-resolution computed tomography. All serological and microbiological tests were negative. The surgical lung biopsy showed fibrinous pleurisy while the search for neoplastic cells resulted negative. Because of symptoms worsening he started low dose steroids without benefits until he died 3 months later for cardiac ischemic attack. We reviewed the literature to identify possible etiologies and a rapidly progressive idiopathic pleurisy revealed to be the most probable diagnosis.

## INTRODUCTION

Here we present a case of idiopathic fibrinous pleurisy affecting a 56-year-old non-smoker male that has shown a rapidly progressive course. Adding a brief review of literature, we discuss the absence of any identified cause of pleurisy as a relatively common condition, requiring attention and clinical awareness.

## CASE REPORT

**Figure 1:**
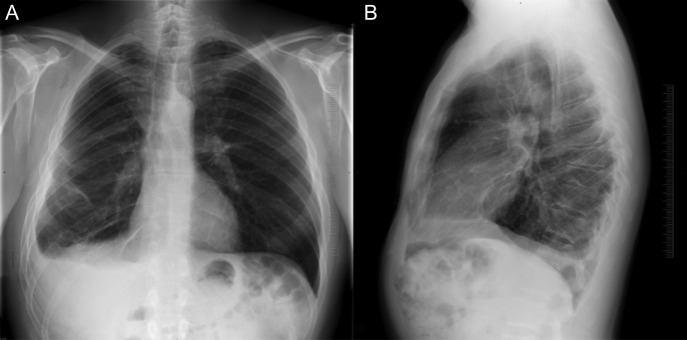
Chest X-ray in antero-posterior (**A**) and lateral view (**B**) showing consistent reduction of the right lung with basal pleural effusion.

**Figure 2:**
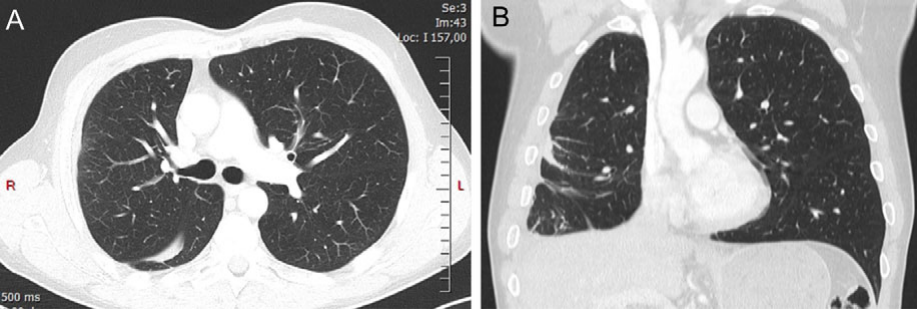
High-resolution CT scan showing right pleural thickening (**A**). Coronal view clearly shows both right lung volume loss and intra-lobar pleural abnormalities (**B**).

**Figure 3:**
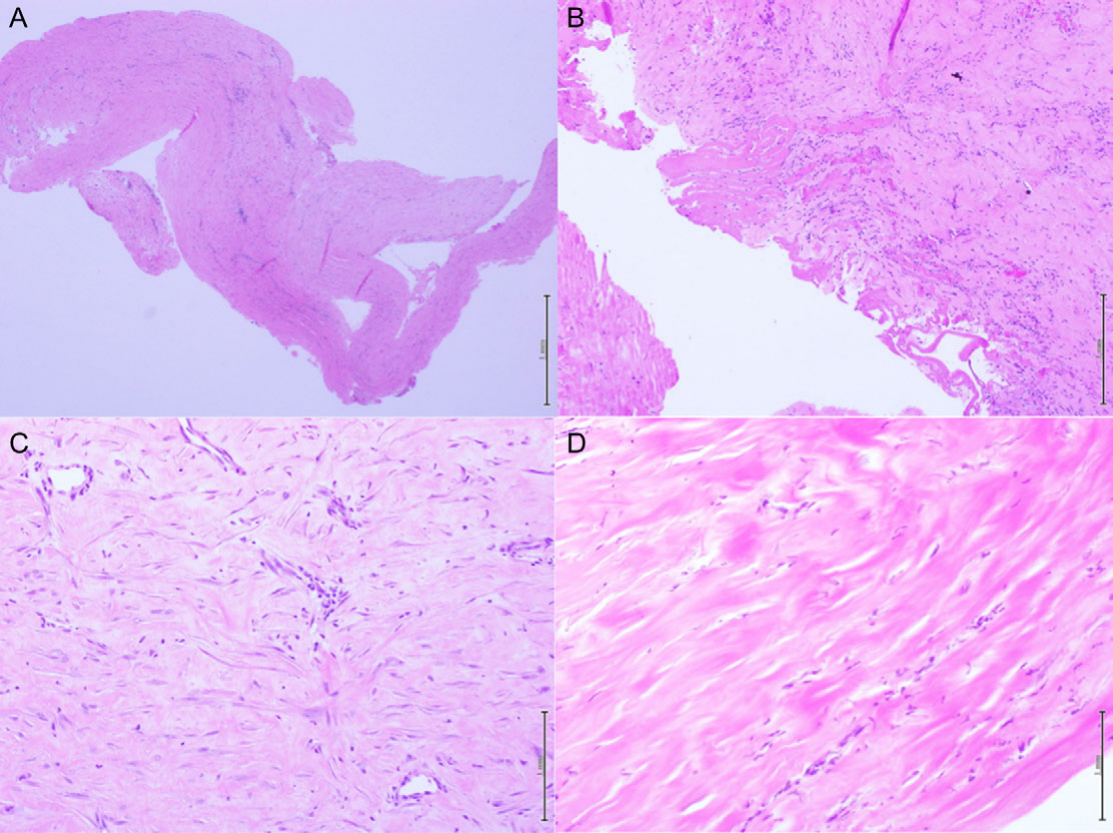
Histologic samples at different magnification of the video assisted pleural biopsy. (**A** and **B**) Show diffuse organized fibrosis with fibrinous exudates and little sporadic amount of inflammatory cells. (**C** and **D**) Show fibroblasts with collagen deposition.

A 56-year-old non-smoker male, engineer for a gas company, presented in early January 2017 with right chest pain and a 1.56 L (29.4%) loss in forced vital capacity (FVC) over the previous 3 years. The anamnestic investigation for professional exposure including asbestos resulted negative. The physical examination revealed the reduction of lung sounds at the right lung basis while no clinical sign of autoimmune disease was found. A chest X-ray (CXR) was performed and showed a considerable reduction in the right lung volume with associated right pleural effusion (Fig. 1). All serological, autoimmune and microbiological tests resulted negative, so he underwent plain and contrast enhanced computed tomography (CT) that revealed a slight reduction in right lung volume with associated right pleural effusion and pleural thickening (Fig. 2). A supplemental investigation was conducted excluding history of trauma, tuberculosis and pneumothorax. Echocardiography excluded pulmonary hypertension. Diaphragm dysfunction was also investigated through phrenic nerve stimulation, but with a negative result. On March 2017 a total body positron emission tomography–computed tomography (PET-CT) with 18-FluoroDeoxyGlucose (18-FDG) was performed and revealed a low intensity hyper-accumulation of 18-FDG in the right pleura with increased concentration in the basal, middle and posterior pleural surface. Thus, a surgical lung biopsy (SLB) was carried, identifying a non-specific fibrinous pleurisy (Fig. 3). The search for neoplastic or infectious cells resulted negative. PFTs performed 1 month later revealed further important drop in FVC (2.5 L loss). The patient developed shortness of breath on exertion, therefore, a prednisolone course (0.25 mg/kg daily) was started. However, PFTs persistently declined with the onset of restrictive respiratory failure. The patient died 2 weeks later for cardiac ischemic attack. Autopsy excluded mesothelioma or other pleural neoplastic diseases, but reported the presence of diffuse fibrinous pleurisy with collagen deposition.

## DISCUSSION

Even after a comprehensive diagnostic work-up, a considerable amount of patients with non-specific pleural exudates do not receive a precise diagnosis [1–3]. The natural history of this frustrating condition still remains poorly understood [1, 4]. With this case report we present the onset and evolution of unilateral idiopathic fibrinous pleurisy, whose description might contribute to better characterize the spectrum presentation of this peculiar condition.

The percentage of patients with persistent pleural effusion in which diagnostic examinations—including VATS biopsy—do not allow the identification of any etiology ranges from 10 to 25% [1–5]. Despite Gunluoglu *et al*. [6] demonstrated that a strict follow-up period should help in formulating the diagnosis suggesting a probable cause of the disease on clinical grounds, the question on the real existence of chronic idiopathic pleurisy still remains [7]. We thus performed a PubMed search using ‘idiopathic’, ‘non-specific’, ‘pleurisy’ and ‘chronic’ as key words and a total amount of 10 works was found. A study by Venekamp *et al*. [8] has shown that benign diseases, in particular heart failure and parapneumonic pleural effusion, were the most frequently identified causes of chronic pleural effusion evolving in fibrosis without no specific histological changes. Different series have identified that pulmonary embolism and drug-related effusion do not lead to definite histological changes in the pleura [5, 8, 9]. In such cases, biopsy samples obtained by VATS are not expected to be diagnostic [8]. Therefore, in these patients a thorough clinical assessment as well as a precise investigation on past medical history and voluptuary habits is mandatory. In particular, autoimmune diseases should be carefully excluded as quite frequent ultimate cause of isolated recurrent pleurisy [5]. An invasive diagnostic procedure, such as the one performed in the presented case, is indicated in cases in which malignancy is suspected and cannot be excluded from a clinical or radiological point of view [10, 11]. When clinical data are silent and alternative causes have been excluded, the identification of non-specific pleurisy obtained on surgical lung specimen should prompt the diagnosis of idiopathic pleuritis and should not be considered a false negative [12].

From data available in literature, the majority of cases presenting with idiopathic pleurisy usually present a benign course (up to 92%) [4, 7, 13]. Recurrence of effusion has been reported in 17% of cases, while complete resolution was described for the great majority of patients (82%) [8]. The clinical presentation of our case seems peculiar due to the rapid progression of the fibrinous process affecting the pleura with dramatic impact on respiratory function tests, namely on FVC. The biopsy proven benign nature of the fibrinous disease was confirmed through autopsy thus excluding neoplastic disease. As we were not able to identify any cause that could be considered responsible for this condition, a diagnosis of idiopathic rapidly progressive pleurisy was made. The uncommon impact on ventilatory capacity of the presented case should be considered in the possible natural history of non-specific pleural disease.
